# LPCAT1, the Enzyme Responsible for Converting LPC to PC, Promotes OPC Differentiation In Vitro

**DOI:** 10.1111/jcmm.70387

**Published:** 2025-01-29

**Authors:** Qi Shang, Xin Zhang, Yingyan Pu, Junjian Lin, Peng Ma, Yuchen Pan, Ming Zhao, Dingya Sun, Li Cao

**Affiliations:** ^1^ Department of Neurobiology, Key Laboratory of Molecular Neurobiology of the Ministry of Education Naval Medical University Shanghai China; ^2^ Beijing Institute of Brain Disorders, Laboratory of Brain Disorders, Ministry of Science and Technology, Collaborative Innovation Center for Brain Disorders Capital Medical University Beijing China; ^3^ Department of Anesthesiology, Changzheng Hospital Naval Medical University Shanghai China; ^4^ Department of Neurology, Naval Specialty Medical Center Naval Medical University Shanghai China

**Keywords:** lipid metabolism, LPCAT1, myelination, OPCs, ZBTB20

## Abstract

Myelin is the key structure for high‐speed information transmission and is formed by oligodendrocytes (OLs) which are differentiated from oligodendrocyte precursor cells (OPCs) in the central nervous system. Lipid is the main component of myelin and the role of lipid metabolism‐related molecules in myelination attach increasing attention. Lysophosphatidylcholine acyltransferase 1 (LPCAT1) mediates the conversion of lysophosphatidylcholine (LPC) to phosphatidylcholine (PC), and its role in myelination draws our interest as LPC is a classical demyelination inducer and PC is a major component of myelin. In this work, LPCAT1 is found expressed in the oligodendrocyte lineage cells during myelination. In vitro experiments showed that the expression level of LPCAT1 gradually increased along with the differentiation process from OPCs to OLs, and over‐expression and interference experiments showed that LPCAT1 promoted OPCs differentiation without affecting their proliferation or apoptosis. Mechanistically, the undertaker of LPCAT1's pro‐differentiation role is not PC, but the phosphorylated mTOR which is a key regulator in OPCs differentiation. RNA sequencing analysis showed LPCAT1 promoted the expression of ZBTB20 which is an important transcription factor related to lipid metabolism and regulates mTOR phosphorylation. In vivo, complex myelin tomacula involving multiple axons was formed after conditionally knocking out LPCAT1 in oligodendrocyte lineage cells, but no obvious myelin thickness abnormalities were observed. Our results indicate that LPCAT1 is an important regulator of myelination, and lipid metabolism‐related molecules may be new valuable targets for the treatment of diseases with myelin abnormalities.

## 1 Introduction

Myelin is an important auxiliary structure for the normal function of axons as it contributes to the rapid transmission of electrical signals and provides protection and nutrition for axons [1]. In the central nervous system (CNS), myelin is formed by oligodendrocytes (OLs) which are differentiated from oligodendrocyte precursor cells (OPCs). During the development of CNS, OPCs undergo proliferation, migration, and eventually spread to the whole CNS [2]. Most OPCs differentiate into OLs finally, and some ones exist stably in undifferentiated state. When demyelination occurs, the undifferentiated OPCs quickly migrate to the lesions, proliferate and differentiate into OLs to remyelinate the demyelinated axons [3]. However, in demyelination‐related neurological diseases such as multiple sclerosis, a large number of OPCs accumulate around demyelinated lesions but fails to differentiate and repair effectively, resulting in the deleterious exposure of axons and irreversible neuronal damage ultimately [4]. Although some molecules regulating differentiation have been found, there is no suitable target for clinical treatment [5, 6]. Therefore, it is of great significance to unearth more molecules participating in differentiation regulation of OPCs.

Lipid is the main component of myelin and can be divided into cholesterol, phospholipid and glycolipid majorly [7]. The three ones account for about 25%, 30% and 40% of the total weight of myelin lipid, respectively [8, 9]. Phospholipid is mainly composed of phosphatidylcholine (PC), phosphatidylethanolamine and sphingomyelin, and glycosphingolipid is the main type of glycolipids. Lipid is not only a constituent of myelin, but also regulates the formation and function of myelin. For example, cholesterol stabilises the myelin‐specific protein PLP [10], and galactosylceramide and sulfatide participate in the transport and stabilisation of NF155 which plays a key role in axon‐glia connection [11]. Disturbed lipid metabolism may cause myelin abnormalities. For example, patients with lipid metabolism disorders such as X‐linked adrenoleukodystrophy and cerebrohepatorenal syndrome often have abnormal myelin structures [12]. Conditional knocking out squalene synthase which is a key enzyme in the synthesis of cholesterol in oligodendrocytes delays the myelination of CNS, reduces the total amount of myelin and harms motor function [13]. Knocking out cerebrosidase sulfotransferase which catalyses the conversion of galactosylceramide to sulfatide leads to myelin injury characterised by structural abnormalities in nodes of Ranvier and paranodal regions [11]. These studies highlight the role of lipid and lipid metabolism‐related molecules in myelin function.

Lysophosphatidylcholine acyltransferase 1 (LPCAT1) is an enzyme expressed in endochylema and its main function is to catalyse lysophosphatidylcholine (LPC) to PC. It can also convert lyso‐platelet activating factor (lyso‐PAF) to platelet activating factor (PAF) which is an ether analogue of PC. The most important recognised function of LPCAT1 is participating in the synthesis of alveolar surfactant dipalmitoylphosphatidylcholine (DPPC) which is pivotal in maintaining the surface tension and ensuring normal breathing [14]. LPCAT1 has also been found playing certain roles in infection [15], diabetes [16] and tumours [17, 18, 19, 20]. In the CNS, mutation of LPCAT1 causes rapid degeneration of rod cells and cone cells, and eventually lead to visual impairment in mouse [21, 22]. It is noteworthy that LPC is a classical demyelination inducer and PC is a major component of myelin, so it may be valuable to explore the function of LPCAT1 in OPCs differentiation which has not been studied.

In the present study, we found that LPCAT1 is highly expressed in oligodendrocyte lineage cells and the expression level increased gradually during the differentiation process. In vitro, cytological experiments showed that LPCAT1 promoted the differentiation of OPCs. Mechanistically, LPCAT1's pro‐differentiation role is not through PC, but ZBTB20, which is an important transcription factor related to lipid metabolism and regulates mTOR phosphorylation. Besides, conditional‐knockout of LPCAT1 in oligodendrocyte lineage cells resulted in the occurrence of myelin tomacula which wrapped multiple axons. Our work indicated that LPCAT1 is an important regulator of OPCs differentiation, which added new evidence for the perspective that lipid metabolism‐related molecules may be new valuable targets for the treatment of diseases with myelin abnormalities.

## 2 Results

### 2.1 The Expression of LPCAT1 Gradually Increased During OPCs Differentiation

**FIGURE 1:**
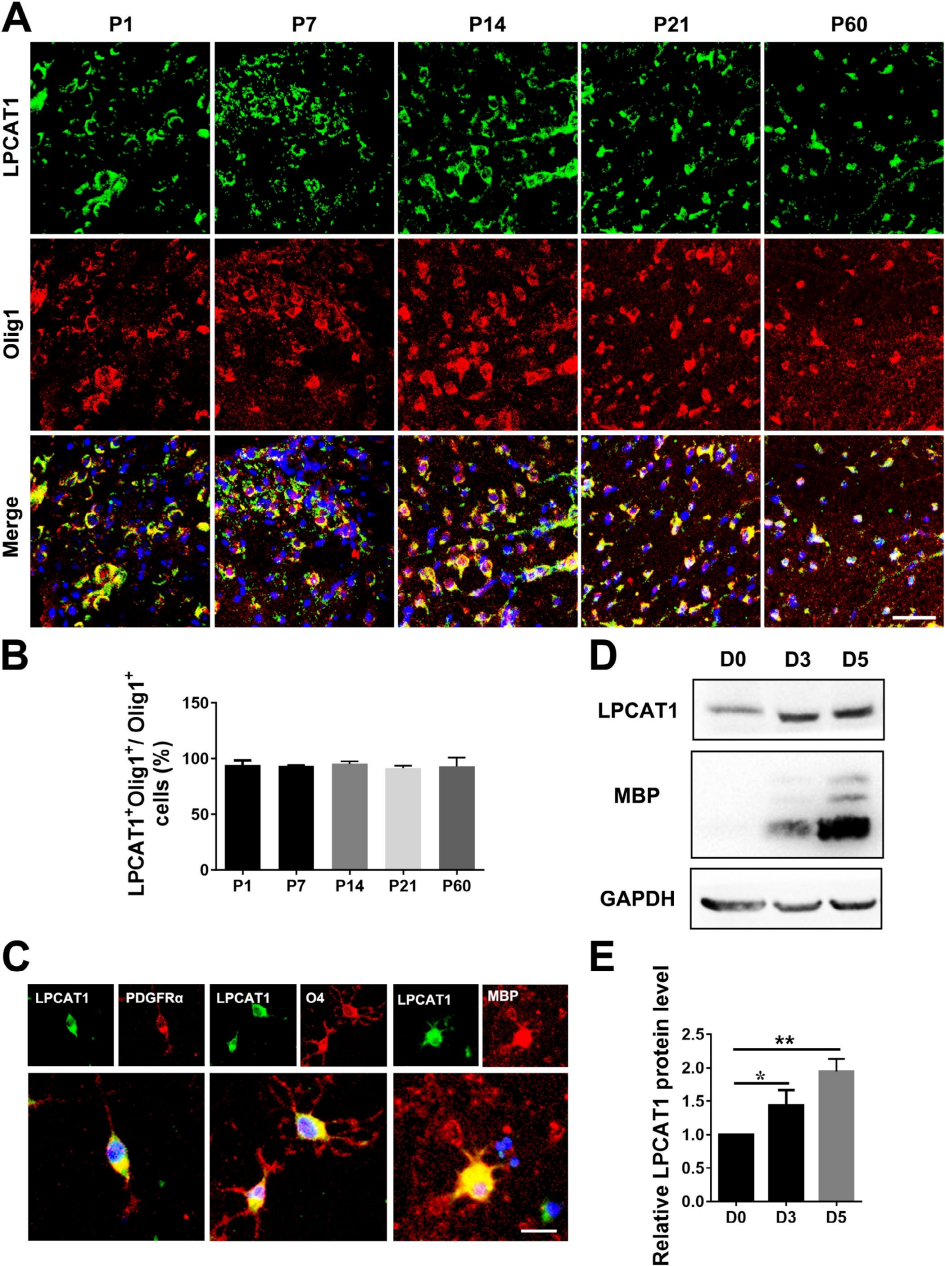
The expression of LPCAT1 is increased during the maturation of OLs. (A, B) Representative immunofluorescence staining (A) and quantitative analysis (B) of LPCAT1 (green) in Olig1 positive oligodendrocyte lineage cells (red) during development. Scale bar = 30 μm. (C) Representative immunofluorescence staining of LPCAT1 (green), PDGFRα (red), O4 (red) and MBP (red) in OPCs at different differentiation stages. Scale bar = 15 μm. (D, E) Representative western blotting (D) and quantitative analysis (E) of LPCAT1 in OPCs at different time points cultured in differentiation medium in vitro. Data are normalised to D0. *N* = 3 independent experiments. *p < 0.05, **p < 0.01 (Student's *t*‐test). Data are shown as the mean ± SD.

To elucidate whether LPCAT1 is involved in myelination, we first examined the expression of LPCAT1 in oligodendrocyte lineage cells. The spinal cords of mice at 1, 7, 14, 21 and 60 day after birth were taken and immunofluorescence staining was done. The oligodendrocyte lineage cells were labelled with Olig1, and the results showed that LPCAT1 and Olig1 co‐labelled cells were found at all time points. It indicates that LPCAT1 is continuously expressed in oligodendrocyte lineage cells during the development of myelin (Figure 1A,B). In order to distinguish the different stages of OPCs differentiation more accurately, we obtained primary cultured rat OPCs and cultured these cells in differentiation medium in vitro. PDGFRα, O4 and MBP antibodies were used to labelled OPCs, differenting OLs and differented OLs, respectively. The immunofluorescence staining showed that LPCAT1 was expressed at all stages of differentiation in vitro (Figure 1C). Further, quantitative analyses using western blotting showed that the expression level of LPCAT1 increased gradually during the differentiation of OPCs (Figure 1D,E), indicating that LPCAT1 may dynamically participate in the differentiation process of OPCs.

### 2.2 LPCAT1 Promotes the Differentiation of OPCs In Vitro

**FIGURE 2:**
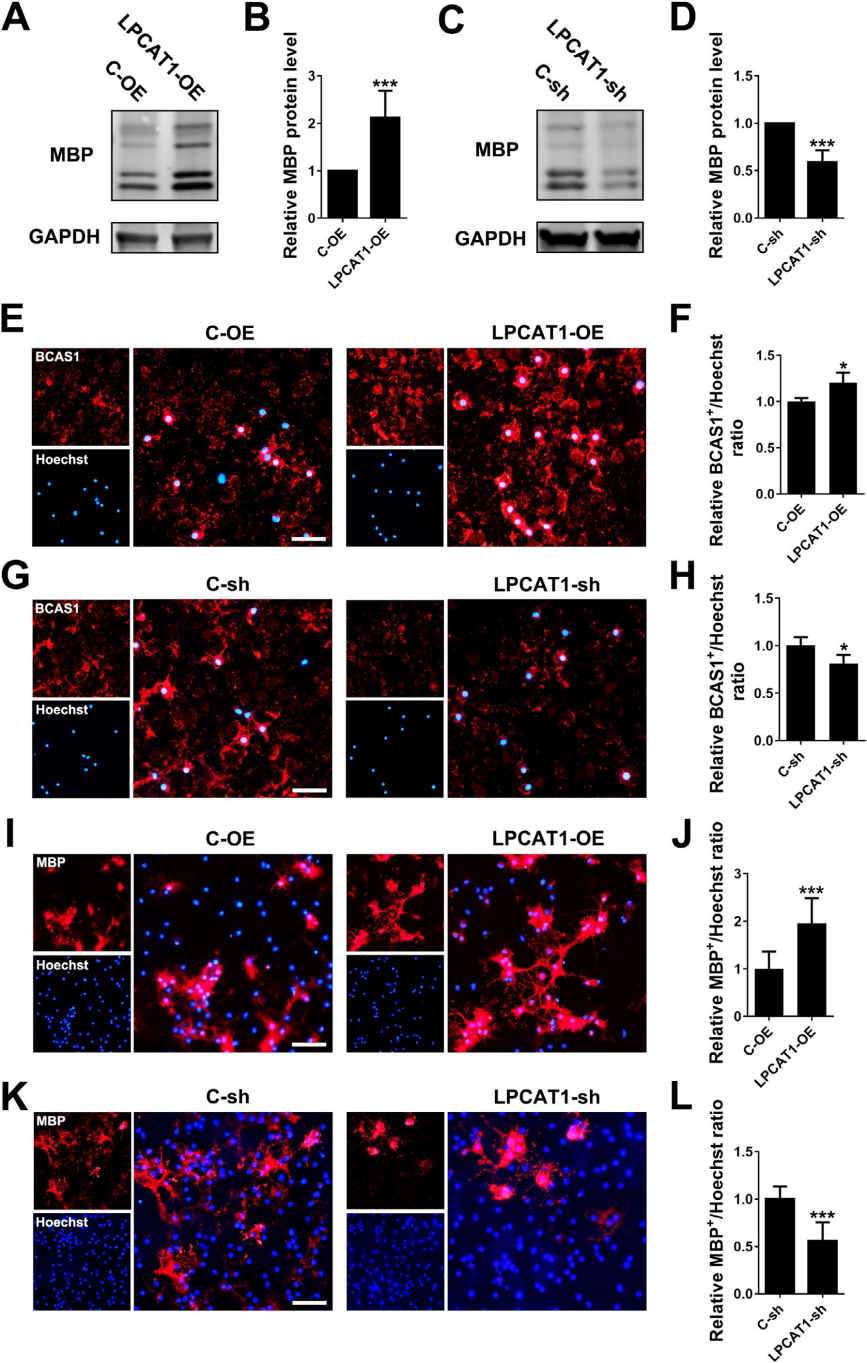
LPCAT1 promotes the differentiation of OPCs in vitro. (A, B) Representative western blotting (A) and quantitative analysis (B) of MBP in OPCs transfected with C‐OE or LPCAT1‐OE lentivirus vectors and differentiated for 48 h. *N* = 7 independent experiments. (C, D) Representative western blotting (C) and quantitative analysis (D) of MBP in OPCs transfected with C‐sh or LPCAT1‐sh lentivirus vectors and differentiated for 48 h. *N* = 6 independent experiments. (E, F) Representative immunofluorescence staining (E) of BCAS1 (red) in OPCs transfected with C‐OE or LPCAT1‐OE lentivirus vectors and differentiated for 48 h. Scale bar = 50 μm. The ratio of BCAS1^+^/Hoechst^+^ cells was calculated and compared between groups (F). *N* = 3 independent experiments. (G, H) Representative immunofluorescence staining (G) of BCAS1 (red) in OPCs transfected with C‐sh or LPCAT1‐sh lentivirus vectors and differentiated for 48 h. Scale bar = 50 μm. The ratio of BCAS1^+^/Hoechst^+^ cells was calculated and compared between groups (H). *N* = 3 independent experiments. (I, J) Representative immunofluorescence staining (I) of MBP (red) in OPCs transfected with C‐OE or LPCAT1‐OE lentivirus vectors and differentiated for 48 h. Scale bar = 50 μm. The ratio of MBP^+^/Hoechst^+^ cells was calculated and compared between groups (J). *N* = 3 independent experiments. (K, L) Representative immunofluorescence staining (K) of MBP (red) in OPCs transfected with C‐sh or LPCAT1‐sh lentivirus vectors and differentiated for 48 h. Scale bar = 50 μm. The ratio of MBP^+^/Hoechst^+^ cells was calculated and compared between groups (L). *N* = 3 independent experiments. *p < 0.05, ***p < 0.001 (Student's *t*‐test). Data are shown as the mean ± SD.

The up‐regulation of LPCAT1 during the differentiation process indicates that LPCAT1 may regulate OPC differentiation. To clarify this question, we constructed LPCAT1 over‐expression and interference lentivirus, and transfected the lentivirus into primary cultured rat OPCs. Using BCAS1 and MBP as indicators of early myelinating or differentiated OLs, respectively, western blotting and immunofluorescence results showed that the MBP expression level and MBP^+^ cells were significantly increased in the LPCAT1 over‐expression group (Figure 2A,B,I,J), and were significantly decreased in the interference group compared to control (Figure 2C,D,K,L), indicating that LPCAT1 indeed promoted the differentiation of OPCs in vitro. The ratio of BCAS1^+^ cells showed a similar pattern of change (Figure 2E–H).

As the proliferation and apoptosis of OPCs have significant influences on differentiation, we further examined the effects of LPCAT1 on the proliferation and apoptosis of OPCs. BrdU was used to label proliferating OPCs, and cleaved Caspase3 was used to characterise apoptotic cells. Immunofluorescence results showed that there were no significant differences in the proportion of GFP^+^BrdU^+^ or GFP^+^Caspase3^+^ double‐positive cells in total GFP^+^ cells among LPCAT1 over‐expressing or interfering groups compared with control (Figure S1), indicating that LPCAT1 did not affect the proliferation or apoptosis of OPCs.

### 2.3 The Pro‐Differentiation Effect of LPCAT1 Is Not Through PC

**FIGURE 3:**
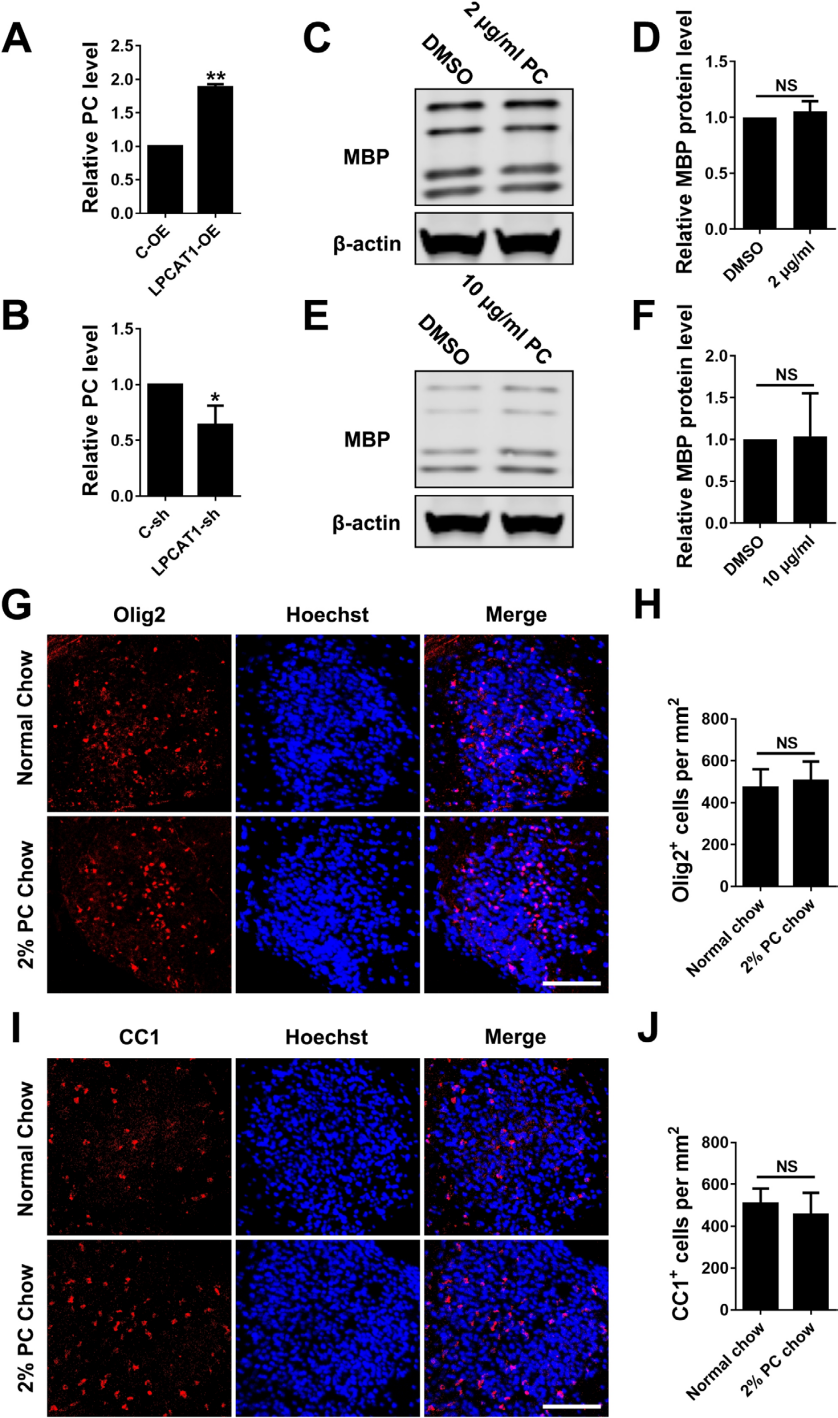
Phosphatidylcholine does not affect OPCs differentiation. (A, B) Relative PC level in OPCs transfected with LPCAT1‐OE (A) or LPCAT1‐sh (B) lentivirus vectors and differentiated for 48 h compared with the control group. *N* = 3 independent experiments. (C, D) Representative western blotting (C) and quantitative analysis (D) of MBP in OPCs added DMSO or 2 μg/mL PC and differentiated for 48 h in vitro. *N* = 3 independent experiments. (E, F) Representative western blotting (E) and quantitative analysis (F) of MBP in OPCs added DMSO or 5 μg/mL PC and differentiated for 48 h in vitro. *N* = 3 independent experiments. (G, H) Representative immunofluorescence staining (G) of Olig2 (red) in demyelinated lesions fed with normal or 2% PC chow at 14 dpi. Scale bar = 100 μm. The density of Olig2^+^ positive cells was calculated and compared between groups (H). *N* = 3 independent experiments. (I, J) Representative immunofluorescence staining (I) of CC1 (red) in demyelinated lesions fed with normal or 2% PC chow at 14 dpi. Scale bar = 100 μm. The density of CC1^+^ positive cells was calculated and compared between groups (J). *N* = 3 independent experiments. *p < 0.05, **p < 0.01 (Student's *t*‐test). Data are shown as the mean ± SD.

The main physiological function of LPCAT1 is to catalyse LPC to PC, and PC is an important component of myelin lipids. Studies have shown that addition of PC can reverse peripheral myelinopathy in Charcot–Marie–Tooth disease 1A (CMT1A) [23]. CMT1A is a peripheral nerve disease with aberrant myelination during postnatal development, followed by slowly progressive demyelination and axonal loss during adult life. But PC has been shown having no obvious effect on the development of normal peripheral myelin. As there has been no research focusing on the effect of PC on the differentiation of OPCs in the CNS, so we examined whether LPCAT1 promoted the differentiation of OPC through producing PC. Firstly, we examined whether over‐expression or interference of LPCAT1 affected PC level in OPCs. The results showed that over‐expressing LPCAT1 indeed increased PC level in OPCs while interfering LPCAT1 decreased the level (Figure 3A,B). Secondly, we added PC of different concentrations to rat primary cultured OPCs to explore the effect of PC on the differentiation of OPCs in vitro. Western blotting results showed that PC did not affect the expression of MBP in differentiated OLs (Figure 3C–F). Finally, we constructed an LPC‐induced mouse spinal cord demyelination model and fed mice with PC‐rich feed. Immunofluorescence staining showed that PC did not affect the genesis of OLs in demyelinating lesions (Figure 3G–I). These results indicated that PC had no obvious effect on the differentiation of OPCs directly and the pro‐differentiation effect of LPCAT1 in OPCs was not through regulating PC production.

### 2.4 LPCAT1 Promotes OPCs Differentiation by Regulating mTOR Signalling Pathways

**FIGURE 4:**
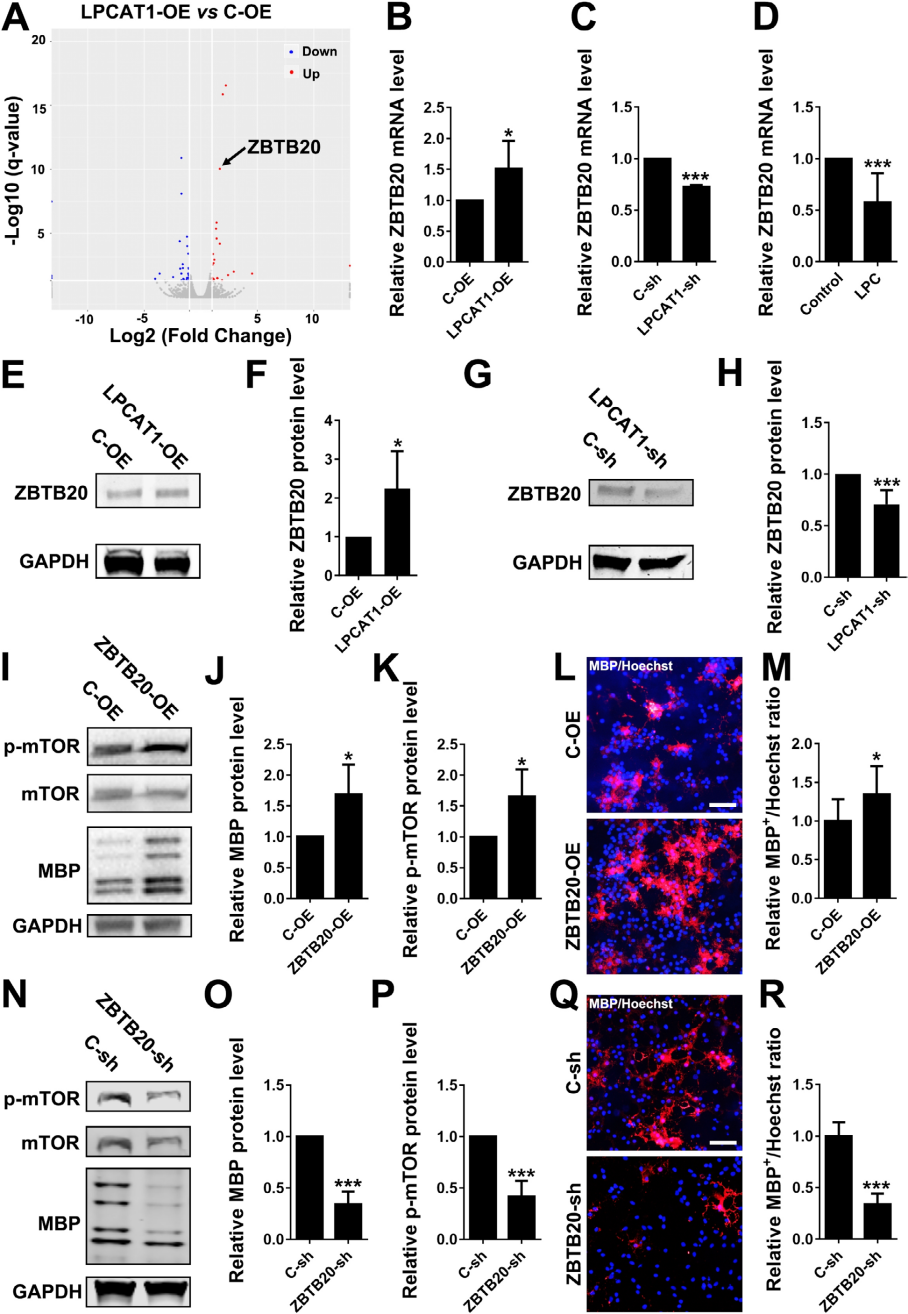
LPCAT1 affects OPCs differentiation by ZBTB20. (A) The volcano map of genes that changed in OPCs transfected with LPCAT1‐OE lentivirus vector compared with OPCs transfected with C‐OE lentivirus vector and differentiated for 48 h. (B, C) Relative level of ZBTB20 in OPCs transfected with LPCAT1‐OE (B) or LPCAT1‐sh (C) lentivirus vectors compared with the control group. *N* = 3 independent experiments. (D) Relative level of ZBTB20 in OPCs added with LPC (20 μg/mL) compared with the control group. *N* = 3 independent experiments. (E, F) Representative western blotting (E) and quantitative analysis (F) of ZBTB20 in OPCs transfected with C‐OE or LPCAT1‐OE lentivirus vectors and differentiated for 48 h. *N* = 4 independent experiments. (G, H) Representative western blotting (G) and quantitative analysis (H) of ZBTB20 in OPCs transfected with C‐sh or LPCAT1‐sh lentivirus vectors and differentiated for 48 h. *N* = 4 independent experiments. (I–K) Representative western blotting (I) and quantitative analysis of MBP (J) and p‐mTOR (K) in OPCs transfected with C‐OE or ZBTB20‐OE lentivirus vectors and differentiated for 48 h. *N* = 4 independent experiments. (L, M) Representative immunofluorescence staining (L) of MBP (red) in OPCs transfected with C‐OE or ZBTB20‐OE lentivirus vectors and differentiated for 48 h. Scale bar = 50 μm. The ratio of MBP^+^/Hoechst^+^ cells was calculated and compared between groups (M). *N* = 3 independent experiments. (N–P) Representative western blotting (N) and quantitative analysis of MBP (O) and p‐mTOR (P) in OPCs transfected with C‐sh or ZBTB20‐sh lentivirus vectors and differentiated for 48 h. *N* = 3 independent experiments. (Q, R) Representative immunofluorescence staining (Q) of MBP (red) in OPCs transfected with C‐sh or ZBTB20‐sh lentivirus vectors and differentiated for 48 h. Scale bar = 50 μm. The ratio of MBP^+^/Hoechst^+^ cells was calculated and compared between groups (R). *N* = 3 independent experiments. *p < 0.05, ***p < 0.001 (Student's *t*‐test). Data are shown as the mean ± SD.

**FIGURE 5:**
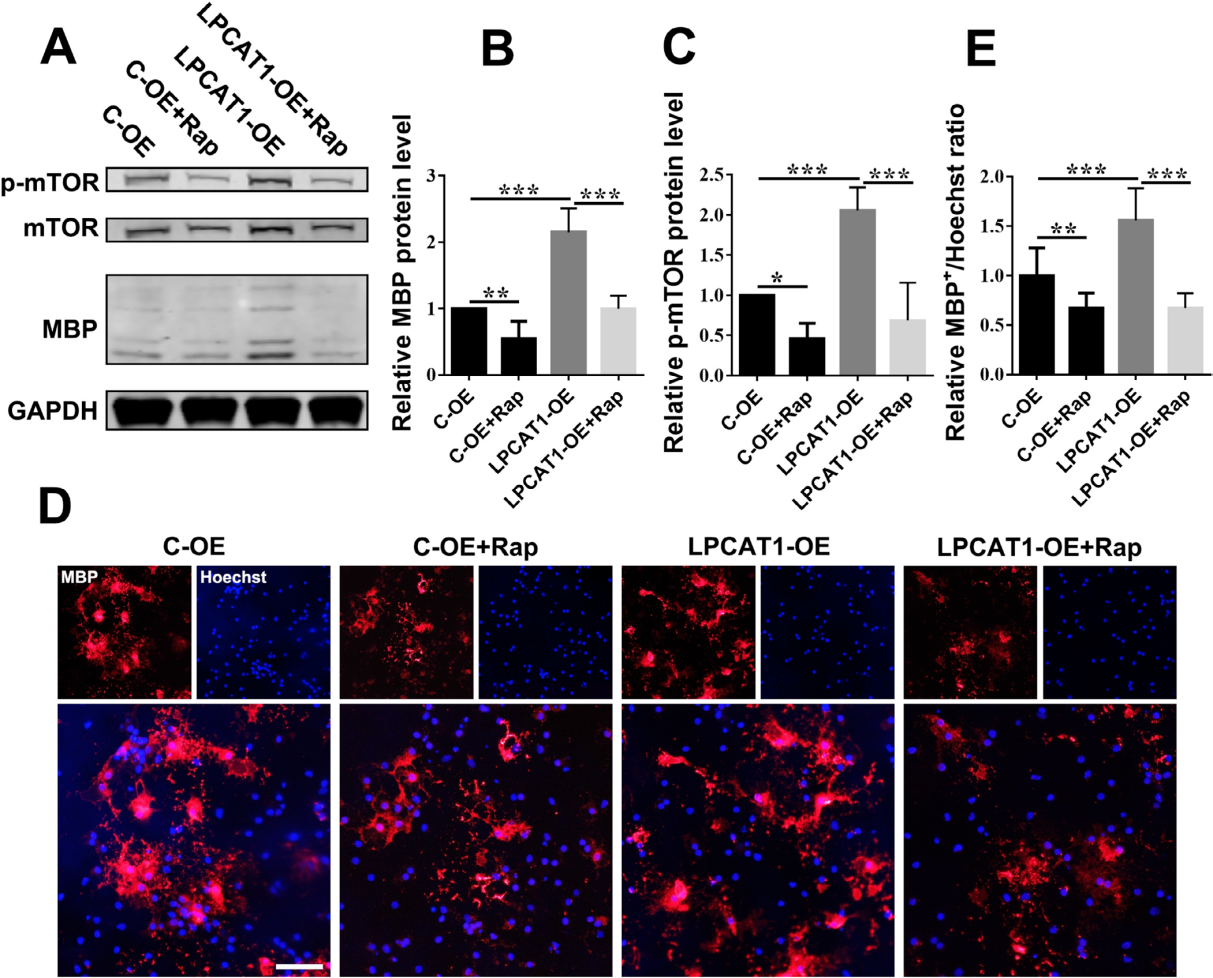
LPCAT1 affects OPCs differentiation by regulating mTOR pathway. (A–C) Representative western blotting (A) and quantitative analysis of MBP (B) and p‐mTOR (C) in OPCs transfected with C‐OE or LPCAT1‐OE lentivirus vectors as well as added rapamycin and differentiated for 48 h. *N* = 3 independent experiments. (D, E) Representative immunofluorescence staining (D) of MBP (red) in OPCs transfected with C‐OE or LPCAT1‐OE lentivirus vectors as well as added rapamycin and differentiated for 48 h. Scale bar = 50 μm. The ratio of MBP^+^/Hoechst^+^ cells was calculated and compared between groups (E). *N* = 3 independent experiments. *p < 0.05, **p < 0.01, ***p < 0.001 (one‐way ANOVA with S‐N‐K's post hoc test). Data are shown as the mean ± SD.

In order to further explore the molecular mechanism of LPCAT1, we performed RNA sequencing analysis using OPCs transfected with LPCAT1 over‐expression lentivirus. The results showed that 22 molecules were significantly up‐regulated and 27 ones were down‐regulated compared to control OPCs (Figure 4A). After taking the expression level and the degree of fold change into consideration, we selected the transcription factor ZBTB20 for further study. ZBTB20 is closely related to lipid metabolism and has been reported regulating the PI3K/AKT/mTOR signalling pathway which is the main pathway regulating the differentiation of OPCs [24]. QPCR and western blotting experiments showed that the expression level of ZBTB20 in OPCs over‐expressing LPCAT1 were significantly higher than those in the control group, and decreased in OPCs interfering with LPCAT1 (Figure 4B,C,E–H). LPC, the substrate of LPCAT1, inhibited the expression of ZBTB20, indicating that LPCAT1 might up‐regulate the expression of ZBTB20 by reducing LPC (Figure 4D). Western blotting showed that over‐expressing ZBTB20 increased the levels of p‐mTOR and MBP (Figure 4I–K), and interfering ZBTB20 down‐regulated the levels of p‐mTOR (Figure 4N–P). MBP^+^ cells were also significantly increased in the ZBTB20 over‐expression group and decreased in the ZBTB20 interfering group (Figure 4L,M,Q,R). Consistently, western blotting showed that over‐expressing LPCAT1 increased the levels of p‐mTOR, and rapamycin, the antagonist of mTOR, blocked the pro‐differentiation effect of LPCAT1 (Figure 5A–C). Immunofluorescence staining showed similar results (Figure 5D,E). These results indicated that LPCAT1 could affect the phosphorylation levels of mTOR by regulating the expression of ZBTB20, thus promoting the differentiation of OPCs.

### 2.5 Knocking Down LPCAT1 in Oligodendrocyte Lineage Cells Does Not Affected Myelination In Vivo

**FIGURE 6:**
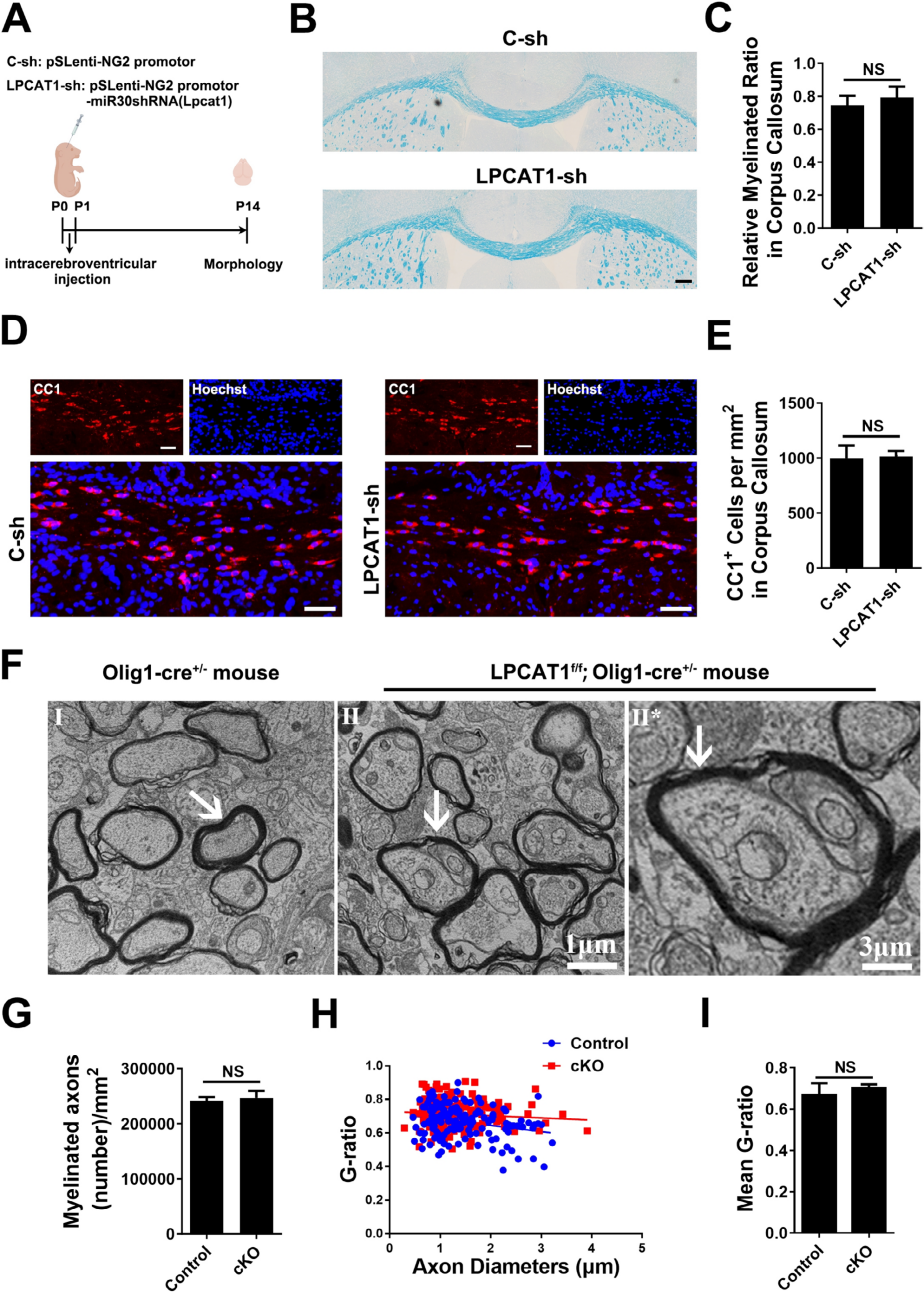
Knocking down LPCAT1 in oligodendrocyte lineage cells does not affected myelination in vivo. (A) A schematic map of experimental timeline and mouse lines resulting in knockdown of LPCAT1 in oligodendrocyte lineage cells. (B, C) Representative Luxol fast blue staining (B) and quantitative analysis of myelinated area (C) in corpus callosum from the mice injected with C‐sh or LPCAT1‐sh lentivirus vectors at P14. Scale bar = 200 μm. *N* = 7 mice per group. (D, E) Representative immunofluorescence staining (D) and quantitative analysis of CC1^+^ OLs (E) in corpus callosum from the mice injected with C‐sh or LPCAT1‐sh lentivirus vectors at P14. Scale bar = 50 μm. *N* = 7 mice per group. (F) Representative electron micrographs of dorsal column spinal cord sections from the conditional knockout or control mice at P14. Scale bar = 1 μm. (G) The density of myelinated axons was calculated and compared between groups. *N* = 3 mice per group. (H) Analysis of the myelinated axons showed no change of the g‐ratio between control group (blue) and conditional knockout mice (red) at P14. *N* = 3 mice per group. (I) Statistical results of G‐ratio between the conditional knockout and control group. *N* = 3 mice per group. NS: *p* > 0.05 (Student's *t*‐test). Data are shown as the mean ± SD.

In order to study the effect of LPCAT1 on myelin development in vivo, we constructed LPCAT1 interference lentivirus driven by NG2 promoter and injected the lentivirus into neonatal mice via the lateral ventricle (Figure 6A). Using SOX10 to label oligodendrocyte lineage cells, the immunofluorescence staining showed that the proportion of LPCAT1^+^SOX10^+^ cells in SOX10^+^ cells decreased significantly in LPCAT1‐sh mice compared to control ones (Figure S3A,B). The peak of myelination in mice is the 14th day after birth (P14), so we chose this time point to study the effect of LPCAT1 on the development of myelin. Luxol fast blue (LFB) and immunofluorescence results showed that the myelinated area and the number of CC1^+^ OLs in corpus callosum were not changed (Figure 6B–E). Besides, we constructed LPCAT1‐loxp mice and bred with Olig1‐cre mice (LPCAT1‐loxp; Olig1‐cre) to conditional knock‐out LPCAT1 in oligodendrocyte lineage cells (Figure S3C–E). Electron microscopy (EM) analysis of the anterior horn of the spinal cord showed that complex myelin tomacula involving multiple axons was formed after conditionally knocking out LPCAT1 in oligodendrocyte lineage cells (Figure 5F), but no obvious changes of myelin thickness or proportion of myelinated axons were observed (Figure 5G–I), indicating that knocking out LPCAT1 in oligodendrocyte lineage cells did not affected myelination, but may hinder the normal myelin‐axon interaction in vivo.

## 3 Discussion

LPCAT1 is a member of the LPCAT family which includes LPCAT2 and LPCAT3 additionally, and all the three ones play roles in lipid metabolism [25]. In recent years, lipids and lipid metabolism related molecules have received increasing attention and have been found playing roles in multiple biological processes and diseases like inflammation and tumours [26, 27]. In this study, we found that LPCAT1 played an important role in the process of central myelination, which promoted OPCs differentiation in vitro and participated in the normal axon wrapping in vivo. However, LPCAT1 did not play its role through regulating PC production, but affected the expression of ZBTB20 thus activated the mTOR signalling pathway. This research uncovers new function and mechanism of LPCAT1, and provides a new target for the regulation of the myelination in the CNS.

In our work, LPCAT1 was found with the ability to influence transcriptome, and this may be through catalysing histone protein O‐palmitoylation to regulate mRNA synthesis as reported [28]. ZBTB20, which is an important transcription factor closely related to lipid metabolism and reported regulating the mTOR signalling pathway, was a representative one affected by LPCAT1. It has been reported that the mTOR signalling pathway can be affected by growth factors, some transcription enhancing elements, and linked cascades like intracellular metabolism. Besides, as an acyltransferase, LPCAT1 can also affect the lipid metabolism balance of OPCs, and thus the mTOR signalling pathway may be feedback‐regulated as it is involved in the lipid metabolism. In addition, there are many other molecules regulated by LPCAT1 as shown in the RNA sequencing, indicating that LPCAT1 may also function in other ways to affect the mTOR signalling pathway. How the lipid metabolism regulates the expression and phosphorylation of mTOR in a feedback way needs further efforts.

In addition to direct effects on transcription, LPCAT1 may also regulate gene expression by reducing its substrate LPC. LPC is a signalling molecule which can activate multiple pathways [29], and LPC had been showed inhibiting the expression of ZBTB20 (Figure 4D). LPC is the most abundant lysophospholipid with relatively high concentration in human blood. In healthy individuals, the plasma level of LPC ranges from 125 to 143 μM [30]. LPC is derived by the cleaving of PC via the action of phospholipase A2 (PLA2) and/or by the transfer of fatty acids to free cholesterol via lecithin‐cholesterol acyltransferase (LCAT). Inflammation and tissue injury lead to an increase in reactive oxygen species (ROS) and promotes the synthesis of LPC. LPC can be converted back to PC by the LPCATs in the presence of Acyl‐CoA. PC mainly exists as a component of the cell membrane and rarely activates signalling pathways directly. In the study, administration of PC did not have a significant effect on myelination. LPC can activate G protein‐coupled receptors (GPCRs), Toll‐like receptors (TLRs), and several ion channels and has emerged as a key contributor to cellular and tissue biology. It can activate the PKC, p38 MAPK, p42 MAPK and JNK pathways, as well as activate c‐jun transcription [31]. LPC shows harmful effects on various cells that include enhancing inflammatory responses, disrupting mitochondrial integrity and inducing apoptosis [32]. In the central nervous system, LPC has been found inducing demyelination in white matter of the spinal cord by activating G protein‐coupled receptor 17 (Gpr17) signalling [33]. GPR17 reduces the intracellular cAMP level and induces pro‐apoptotic gene XIAP‐associated factor 1 (Xaf1) expression, which in turn inhibits oligodendrocyte survival and precursor cell differentiation [33]. The detailed pathway through which LPC affects ZBTB20 expression requires further exploration.

Our immunofluorescence staining showed that LPCAT1 was widely expressed in the CNS with no specific cell type tropism (Figure S2). This may be the reason why no significant differences of LPCAT1 expression in the mouse corpus callosum were found using western blotting at different stages of myelination (data not shown). The expression pattern of LPCAT1 suggests that it may play multiple roles in the CNS. For example, ZBTB20 which has been found regulated by LPCAT1 is an important regulator of neural cell development. Knocking out ZBTB20 leads to dysplasia of astrocytes and structural abnormality of dendritic spines and branches in neurons, which finally affects learning, memory and behaviour. The expression pattern of LPCAT1 limits its application in the treatment of demyelinating diseases such as MS, but further studies of LPCAT1's functions in other neural cells may make it feasible.

Although LPCAT1 promoted the differentiation of OPCs in vitro, no differences of myelinated area or the number of CC1^+^ OLs were observed in corpus callosum of mice that knocking down LPCAT1 in oligodendrocyte lineage cells at P14. This suggested that the role of LPCAT1 in myelination could be compensated by other molecules in vivo. Interestingly, complex myelin tomacula involving multiple axons was formed in conditional knock‐out mice, showing that the interaction between myelin and axons were disturbed. Similar phenomena can be found in Charcot–Marie–Tooth disease which affects the peripheral nervous system and is caused by abnormalities in cell surface adhesion molecules like CADM4 and cytoskeletal proteins like FGD4 [34]. In the RNA sequencing analysis, the transcription of some molecules related to cell adhesion and cytoskeleton were found regulated by LPCAT1, which may be the reason of the abnormal phenomenon in vivo. This needs further confirmation.

In conclusion, through a series of experiments, we proved that the lipid metabolism‐related molecule LPCAT1 plays an important role in the differentiation of OPCs in vitro and the normal myelination of axons in vivo. This research not only sheds more light on the importance of molecules related to lipid metabolism, but also suggests that the non‐lipid‐dependent manner may be an important functioning way for lipid metabolism‐related molecules, which may be an interesting research field in the future.

## 4 Materials and Methods

### 4.1 Animal experiments

The animal experiments in this study were carried out in adherence with the *National Institutes of Health Guidelines on the Use of Laboratory Animals* and approved by the Naval Medical University Committee on Animal Care. Intracerebroventricular injection was conducted as described by Ji‐Yoen Kim et al. (JOVE 51863). Focal demyelination in dorsal spinal cord induced by L‐a‐lysophosphatidylcholine (LPC; Lysolecithin; Sigma Aldrich) was performed as described before [35]. Briefly, 1 μL of 1% LPC in 0.9% sodium chloride solution was microinjected into the dorsal column at T11–T12 spinal cord of C57/BL6 mice (8–10 weeks) using a microinjector (Hamilton, NE, USA) after the exposure of vertebrae. The day of LPC injection was designated as day 0 (0 dpi). Control mice were injected with 1 μL of saline solution.

### 4.2 Cell Cultures

Primary cortical OPCs were prepared according to our previous work [36]. In brief, mixed cortical glial cell cultures were generated from P0 rat cerebra and were cultured in DMEM containing 10% FBS at 37°C with 5% CO_2_. The medium is changed every 3 days. The flasks were shaken for 1 h on an orbital shaker (180 rpm) 10 days later to remove microglia, followed by an additional 16 h at 200 rpm with fresh medium at 37°C. The purified OPCs were collected from mixed glia followed by leaving cell suspension adhere in uncoated Petri dishes. Then they were seeded at 50,000–5000 cells/cm^2^ on coverslips that were coated with poly‐D‐lysine 1 day before. To examine differentiation, OPCs were cultured in neurobasal medium supplemented with 2% B27 (NB/B27). To expand the OPCs and keep them undifferentiated, culture medium was supplemented with 30% B104 conditioned media.

### 4.3 Lentivirus Transduction

For lentivirus transduction in primary cultured rat OPCs, the target sequence of the LPCAT1 siRNA is “GACCGACTTGTTCCAGGCTAT”. It was ligated into the GV493 plasmid (GeneChem, Shanghai, China). The LPCAT1 over‐expressing lentivirus was also constructed by GeneChem. The titre of concentrated viral particles was 0.5–1 × 10^9^ transducing units/mL. Lentiviral particles were added to cultured oligodendrocyte at a multiplicity of infection (MOI) = 3 and the supernatant was changed 12 h after infection. For intracerebroventricular injection, the target sequence was ligated into the H21315 plasmid which is driven by the NG2 promoter (Obio, Shanghai, China). 2 μL (about 1 × 10^6^ transducing units) was injected into each lateral ventricle.

### 4.4 Immunocytofluorescence and Immunohistofluorescencestaining

Cells on the coverslips were fixed with 4% paraformaldehyde (PFA) for 15 min followed by three washes in PBS at room temperature. Then cells were incubated with primary antibodies for overnight at 4°C as follows: mouse anti‐MBP (Chemicon, MAB382, 1:50), rabbit anti‐BCAS1 (Synaptic Systems, 445,003, 1:50), mouse anti‐cleaved Caspase3 (Milipore, AB3623, 1:100), anti‐PDGFRa (RD systems, AF1062, 1:50), anti‐O4 (Sigma,1:100), anti‐LPCAT1 (Invitrogen, 1:100). Cells then were incubated in FITC‐ or TRITC‐conjugated secondary antibodies (Jackson ImmunoResearch, 1:100) containing Hoechst for 2 h at room temperature. Cells were counted in at least three randomly selected fields from one coverslip and at least three coverslips for each group were counted.

Animals were anaesthetised and perfused with 4% PFA via transcardiac perfusion. Tissues were embedded using OCT (SAKURA) at −20°C. Frozen tissue samples were prepared into 14 μm cryostat sections for further histological analysis. Sections were boiled for 15 min in 10 mM citrate buffer (pH 6.0) at 95°C, then placed in blocking solution, and incubated overnight at 4°Cwith primary antibodies, including anti‐PDGFRα (RD systems, AF1062, 1:50), anti‐CC1 (Milipore, MABC200, 1:100), anti‐Olig1 (Milipore, MAB5540, 1:300), anti‐NeuN (Milipore, MAB377, 1:50), goat anti‐Iba1 (Abcam, ab5076, 1:50), anti‐GFAP (Sigma, G3893, 1:50), anti‐SOX10 (Sigma, 1:100), and anti‐olig2 (Sigma, 1:100). Tissues then were incubated in FITC‐ or TRITC‐conjugated secondary antibodies (Jackson ImmunoResearch, 1:100) containing Hoechst for 2 h at room temperature. The samples were examined by confocal microscopy (SP5 or SP8, Leica). Three spinal cord sections were selected for each sample, and 3–5 mice were tested in each experimental group.

### 4.5 RNA Isolation and qPCR Analysis

Cells were lysed with TRIzol reagent (Invitrogen) and total RNA was extracted according to the manufacturer's instructions and cDNA was synthesised using a RevertAid First Strand cDNA Synthesis Kit (Thermo Scientific Fermentas). The mRNA level was then detected using real‐time quantitative PCR (qPCR). It was performed with the SYBR Green Real‐time PCR Mater Mix (TOYOBO). Gene expression was normalised to a standard housekeeping gene GAPDH using the ΔΔCT method. PCRs were performed under the following conditions: 95°C for 3 min followed by 45 cycles at 95°C for 10 s and 60°C for 30 s. The primers used for LPCAT1 were F: CAAACCACTCACCCGAATGTCC, R: CACGAGGTTCTCTGTCAACTTGC. The primers used for GAPDH were F: CCATCAACGACCCCTTCATT, R: ATTCTCAGCCTTGACTGTGC. The primers used for MBP were F: CTTGTTAATCCGTTCTAATTCCG, R: TTCTGGAAGTTTCGTCCCT. The primers used for ZBTB20 were F:CAAATCGAGCAGTTCAACGACCA, R:CTTAAATTCTAGTGCCATAGCTTG.

### 4.6 Western Blotting Analysis

Western blotting is done in accordance with standard protocols. Primary antibodies include: anti‐LPCAT1 (Proteintech, 1:400), anti‐MBP (Chemicon, MAB382, 1:500), anti‐mTOR (CST, 1:1000), anti‐p‐mTOR (CST, 1:1000), and anti‐gapdh (Kangcheng Biotechnology, 1:10000). Primary antibodies were incubated overnight at 4°C. HRP conjugated secondary antibodies (Kangcheng Biotechnology, 1:3000) or IRDye 680/899 conjugated secondary antibodies (LI‐COR, 1:10000) were incubated for 2 h at room temperature. The protein bands were analysed and quantified using Image Lab analysis (Bio‐Rad) or Image Studio (LI‐COR).

### 4.7 BrdU Incorporation

To detect proliferation of OPCs, BrdU (5‐Bromo‐2‐deoxyUridine, Sigma; 10uM) was added to medium for 6 h before the cells were fixed in 4% PFA. The coverlips were then punched with 0.3% Triton for 10 min, then incubated in 2 N HCl for 30 min and incubated in 0.1 M borate buffer (pH 8.0) for 20 min. The coverslips were then blocked in PBS containing 5% goat serum for 1 h at room temperature and incubated overnight with anti‐BrdU antibody (Sigma, 1: 100) and anti‐GFP (Millipore, 1:300) antibody. Cells then incubated in FITC‐ or TRITC‐conjugated secondary antibodies (Jackson ImmunoResearch, 1:100) for 2 h at room temperature. The percentage of BrdU^+^GFP^+^ cells versus GFP^+^ cells was calculated.

### 4.8 Transmission Electron Microscopy

Briefly, mice were sacrificed and transcardially perfused with 4% PFA. Then, the ventral columns of spinal cords were isolated and fixed in 2.5% glutaraldehyde for 2 h and post‐fixed in 1% osmium tetroxide for 45 min before being dehydrated and embedded in araldite resin. Ultrathin sections (60 nm) were stained in uranyl acetate and lead citrate. Samples were visualised using a transmission electron microscope (Hitachi H‐7650, Japan) at 100 KV.

### 4.9 Statistical Analysis

A two‐tailed Student's *t*‐test was applied for statistical comparison of two groups, and a one‐way ANOVA with S‐N‐K's *post hoc* test was used for multiple groups. The data are presented as the mean ± SD which generally represents biological replicates. The value of *p* < 0.05 was considered statistically significant.

## Author Contributions

**Qi Shang:** conceptualization (equal), investigation (lead). **Xin Zhang:** data curation (equal), formal analysis (lead), methodology (equal). **Yingyan Pu:** investigation (equal), project administration (equal). **Junjian Lin:** investigation (equal), software (equal). **Peng Ma:** resources (equal), supervision (equal). **Yuchen Pan:** supervision (equal), visualization (equal). **Ming Zhao:** funding acquisition (equal), visualization (equal). **Dingya Sun:** writing – original draft (lead), writing – review and editing (supporting). **Li Cao:** funding acquisition (equal), visualization (equal), writing – review and editing (equal).

## Conflicts of Interest

The authors declare no competing financial interests.

## Supporting information


Appendix S1.

## Data Availability

The data that support the findings of this study are available from the corresponding author upon reasonable request.
