# Supplementary material for: LPCAT1, the Enzyme Responsible for Converting LPC to PC, Promotes OPC Differentiation In Vitro
Source: J Cell Mol Med. 2025 Jan 29;29(3):e70387. doi: 10.1111/jcmm.70387 (PMC11775935; doi:10.1111/jcmm.70387)
Supplement: Supplementary file 1 — Appendix S1. [file JCMM-29-e70387-s001.docx]

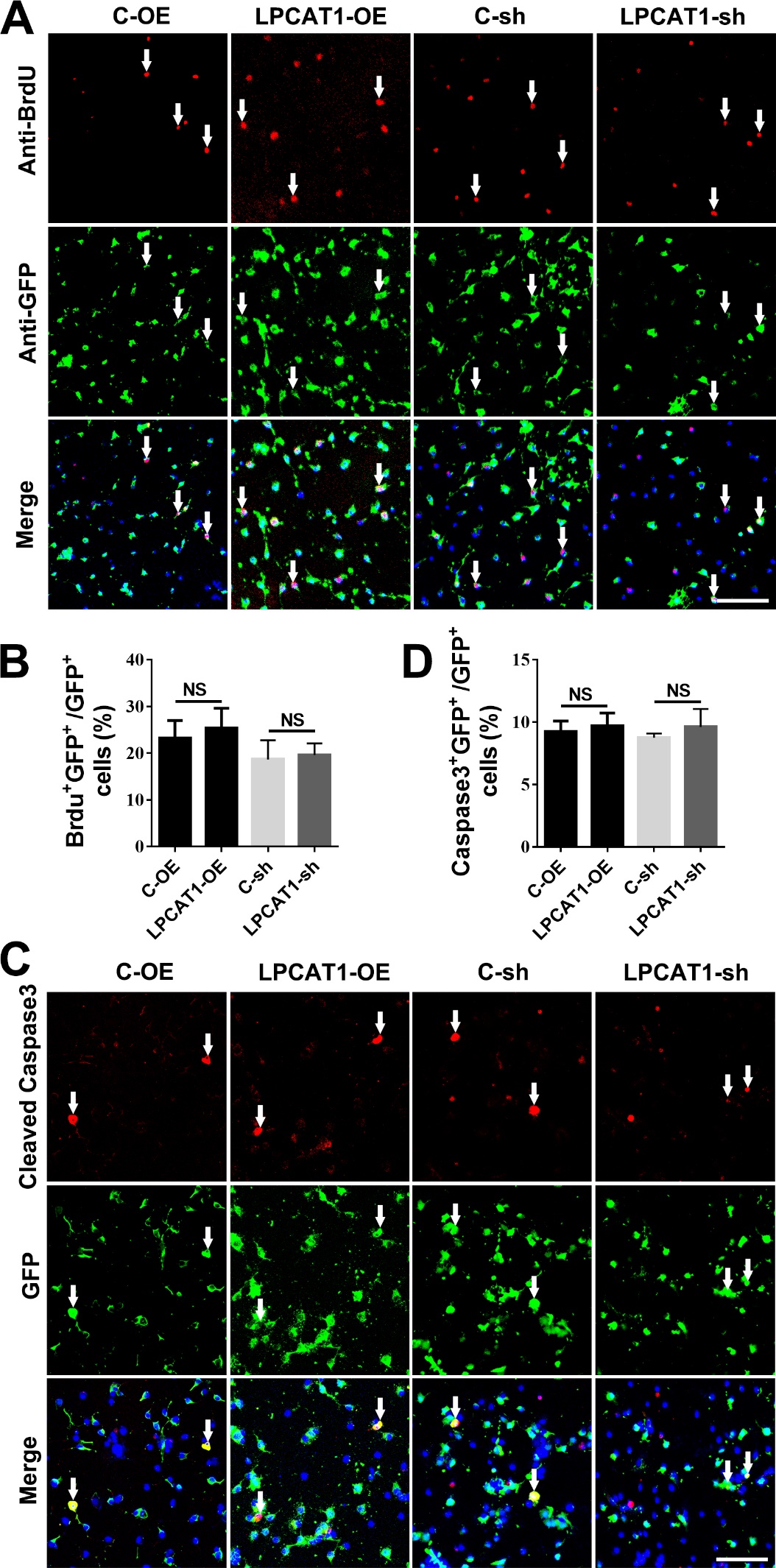


**FIGURE S1：**LPCAT1 does not affect the proliferation or apoptosis of OPCs.

(A, B) Representative immunofluorescence staining (A) of BrdU (red) in OPCs transfected with LPCAT1-OE (left) or LPCAT1-sh (right) lentivirus vectors compared with the control group. Scale bar = 100 μm. The ratio of BrdU^+^GFP^+^/ GFP^+^ cells was calculated and compared between groups (B). N = 3 independent experiments. (C, D) Representative immunofluorescence staining (C) of cleaved caspase3 (red) in OPCs transfected with LPCAT1-OE (left) or LPCAT1-sh (right) lentivirus vectors compared with the control group. Scale bar = 100 μm. The ratio of Caspase3^+^GFP^+^/ GFP^+^ cells was calculated and compared between groups (D). N = 3 independent experiments. Student’s *t*-test. Data are shown as the mean ± SD.


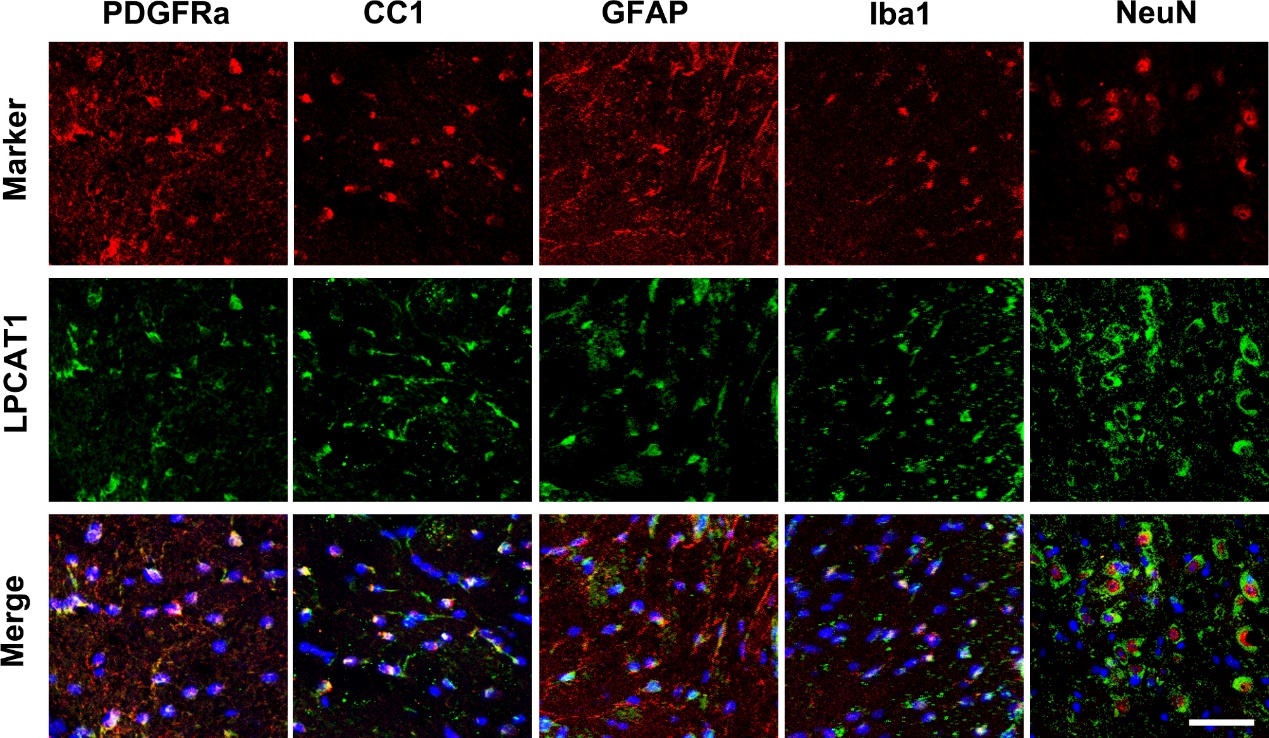


**FIGURE S2：**LPCAT1 is widely expressed in the brain.

Representative immunofluorescence staining of LPCAT1 (green) in OPCs (PDGFRα, red), OLs (CC1, red), Astrocytes (GFAP, red), Microglia (Iba1, red) and Neuron (NeuN, red). Scale bar = 50 μm.


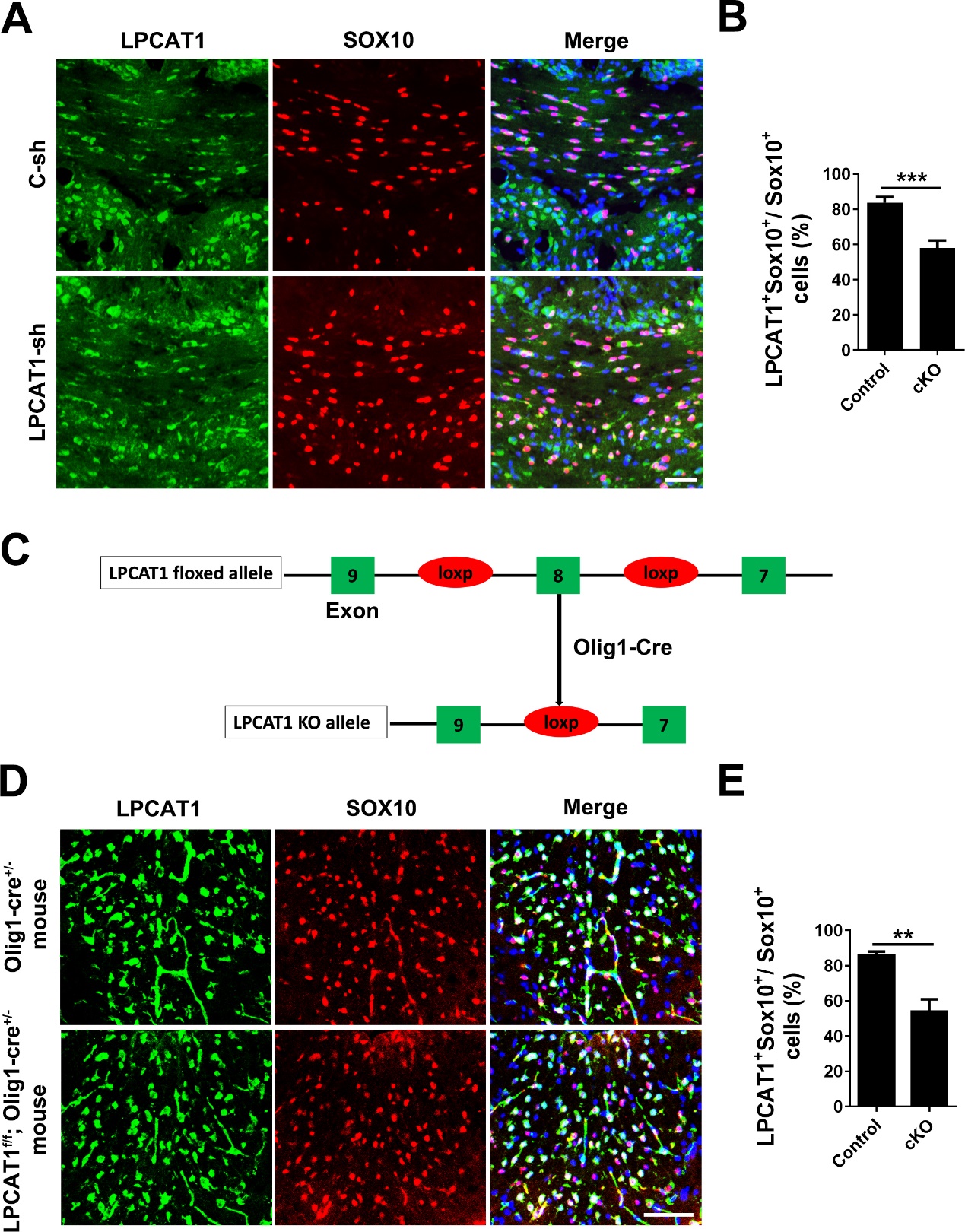


**FIGURE S3：**Validation of the effectiveness of knocking down LPCAT1 or the construction of conditional knock-out mice.

(A, B) Representative immunofluorescence staining (A) of LPCAT1 (green) in SOX10 positive oligodendrocyte lineage cells (red) (B) in corpus callosum from the mice injected with C-sh or LPCAT1-sh lentivirus vectors at P14. Scale bar = 50 µm. N = 3 mice per group. (C) A schematic map of the generation of the mice that conditional knockout of LPCAT1 in oligodendrocyte lineage cells. (D, E) Representative immunofluorescence staining (D) of LPCAT1 (green) in SOX10 positive oligodendrocyte lineage cells (red) in conditional knockout (**LPCAT1^f/f^; Olig1-cre^+/-^**) or control (Olig1-cre^+/-^) mice at P14. Scale bar = 30 μm. The ratio of LPCAT1^+^Sox10^+^/ Sox10^+^ cells was calculated and compared between groups (E). N = 3 mice per group.
